# SENSORY IMPAIRMENT, COGNITIVE FUNCTION, AND DEPRESSION AMONG CHINESE OLDER ADULTS

**DOI:** 10.1093/geroni/igac059.2263

**Published:** 2022-12-20

**Authors:** Shu Xu, YanJhu Su

**Affiliations:** University of Massachusetts Boston, Boston, Massachusetts, United States; University of Massachusetts Boston, Boston, Massachusetts, United States

## Abstract

Sensory impairment (SI) is a contributor to poor mental health and cognitive decline for older adults, and the likelihood of having sensory impairment increases with age. However, the association between sensory impairment and cognition is still under-investigated and the potential mechanisms for the SI-cognition link is still not clear. This study examines the relationships between sensory impairment, depression, and cognitive function among older adults in China. Using nationally representative data from the China Health and Retirement Longitudinal Study 2018, we conducted cross-sectional analysis on adults age 60 years and older (n=7,026). Sensory impairment is defined as having vision impairment (VI) only or hearing impairment (HI) only or dual sensory impairment (DSI). Cognitive function was measured by the Mini-Mental State Examination and depression were assessed by the 10-item Center for Epidemiologic Studies Depression scale. Descriptive analysis showed that 10.66% of older adults experienced sensory impairment. Linear regression analyses revealed that HI and DSI were associated with cognitive declines among older Chinese adults (HI: β=-0.75, p<.01; DSI: β=-1.45, p<.01). SEM results showed that depression partially mediate the relationship between SI and cognition. Sensory-impaired older adults were more likely to have depression (HI: β=1.71, p<.001; DSI: β=4.76, p<.001), which lead to worse cognitive function (HI: β=-1.09, p<.001; DSI: β=-2.80, p<.001). Models were controlled for age, gender, education, social activities, and other covariates. Findings suggest that Chinese older adults experiencing sensory loss are at greater risk of cognitive function declining and that depression play an important role in the relationship between SI and cognition.

